# Black “Reading the Mind in the Eyes” task: The development of a task assessing mentalizing from black faces

**DOI:** 10.1371/journal.pone.0221867

**Published:** 2019-09-19

**Authors:** Grace Handley, Jennifer T. Kubota, Tianyi Li, Jasmin Cloutier

**Affiliations:** 1 Department of Psychological and Brain Sciences, University of Delaware, Newark, Delaware, United States of America; 2 Department of Political Science & International Relations, University of Delaware, Newark, Delaware, United States of America; 3 College of Business Administration, University of Illinois Chicago, Chicago, Illinois, United States of America; York University, CANADA

## Abstract

Researchers investigating various facets of theory of mind, sometimes referred to as mentalizing, are increasingly exploring how social group membership influences this process. To facilitate this research, we introduce the Black Reading the Mind in The Eyes task, a freely available 36-item Black RME task with an array of norming data about these stimuli. Stimuli have been created and equated to match the original Reading the Mind in the Eyes (RME) task which included only White faces. Norming data were collected in three waves that characterized the physical properties of the stimuli and also participants’ subjective ratings of the stimuli. Between each round of ratings, stimuli that did not equate with the original RME task or were not distinctly recognized as Black were removed and new stimuli were incorporated in the next round until we obtained 36 distinctive Black RME targets that matched the 36 mental states used in the original RME stimulus set. Both stimulus sets were similarly difficult and subsequent testing showed that neither Black nor White participants’ mentalizing accuracy varied as a function of target race. We provide instructions for obtaining the database and stimulus ratings.

## Introduction

The ability to infer others’ mental states, or mentalize, is a critical cognitive skill that enables people to successfully navigate an increasingly complex social world. Social categories such as race importantly shape the ways in which humans perceive and evaluate each other; however, one of the most oft-cited measures of mentalizing from visual facial cues, the Reading the Mind in the Eyes (RME) task, uses exclusively White target faces [1]. To date, few studies have addressed how mental states are inferred from non-White faces, and whether or not this differs from how mental states are inferred from White faces (c.f., Adams et al., 2009) [2]. To address this gap, we developed a version of the RME task that uses Black target faces and is compatible with the original Baron-Cohen et al. (2001) stimuli [1]. The goal of this database is to provide an open access stimulus set to spur future research, and we intend to expand the stimulus set in the future to include other race faces. In this manuscript, we provide researchers with equating information and a link to the open access directory for the Black RME task.

People can rapidly and (somewhat) accurately extract rich social information from limited perceptual cues [3–8]. Information about others’ mental states is among the most important social information that can be gleaned from such cues. Deficits in mentalizing are associated with difficulty in understanding and predicting the social environment and are suggested to be a major feature of autism spectrum disorders [9]. The eyes in particular have been shown to have a uniquely important role in conveying nuanced social information, particularly information pertaining to secondary human emotions (e.g., [10–18]). Therefore, the RME test was developed as a measure of peoples’ ability to infer mental states from the eyes.

### The RME task

The original RME task was developed by Baron-Cohen and colleagues in 1997, then revised in 2001; this revised test has since been widely used to assess mentalizing ability [1,11]. Baron-Cohen and colleagues initially found that being shown the eyes alone produced equally accurate judgments of mental states (e.g. thoughtful, suspicious) as being shown an entire face [1]. Therefore, in the RME task, participants view a cropped photograph of a set of eyes that portray a complex mental state and then select the correct mental state from four possible options. Researchers quantify the degree to which individuals correctly identify the mental state for each set of eyes, yielding a score of mentalizing accuracy. Among other questions, social cognition researchers have used this task to measure peoples’ tendency to engage in spontaneous mental state inferences (e.g., [19,20]). The RME task has specifically been favored as a test of adult mentalizing ability because tasks utilized with children are often too easy for adults and do not allow for meaningful assessment of mentalizing *accuracy* in adulthood; that is, neurotypical adults tend to perform at ceiling on standard false belief tasks when not under cognitive load [21,22]. Given the multitude of ways that face processing and social cognition can vary as a function of perceptual target race, it is paramount to expand our understanding of mentalizing processes by including more diverse face stimuli. The little research that has been done on cross-race mentalizing indicates it very well may differ as a function of target race [2]. The present work therefore aims to develop and share access to an RME test that uses Black stimuli but is otherwise well equated with the widely-used original stimulus set.

### Differences in social cognition as a function of target race

Particularly relevant to the present work, Adams and colleagues (2009) tested a cross-cultural version of the RME test using White and East Asian eyes with native White American and native Japanese participants [2]. They found evidence for both behavioral and neural differences in same- versus cross-race mentalizing: participants were more accurate at inferring mental states from same-race targets than from cross-race targets, and this difference was associated with increased bilateral posterior superior temporal sulci (STS) recruitment during mentalizing [2]. This region has been extensively implicated in neuroimaging studies of theory of mind, particularly among studies using the RME test (for reviews, see [23–25]).

Additionally, there is strong evidence that face processing and various social cognitive processes may vary as a function of perceptual target race. The Other-Race Effect is a particularly robust example of how face processing varies across target race; people consistently show worse recognition memory for other-race faces compared to same-race faces ([26,27]; for review, see [28]). Other-race faces are also thought to be processed in a more piecemeal inexpert manner based on facial features, unlike own-race faces which typically are processed in a configural, expert way [29,30]. Research suggests that piecemeal face processing may be a suboptimal strategy; such processing has been associated with decreased recognition memory [31–33], and has recently even been linked to diminished perceptions of a face’s humanness [34]. Numerous other social cognitive processes vary across perceptual target race, including: stereotype activation [35–38], implicit racial attitudes and affective bias [39–44], trustworthiness judgments and decision-making [45–47], and the neural empathic response to pain [48,49]. These intergroup differences in perception and evaluation can have a host of negative consequences for intergroup relations. However, in contrast to these other areas of investigation, intergroup mentalizing, a fundamental aspect of intergroup relations and social interactions, has yet to receive substantial research attention. One possible explanation for this gap in the literature is the lack of available measures.

### Current research

To facilitate research on intergroup mentalizing, we sought to develop and share access to a 36-item Black RME (BRME) task that was equated with the original 36-item White RME task. We present two studies that describe stimulus selection for the BRME (Study 1) and a comparison of Black and White perceivers’ performance on both the original RME and the BRME (Study 2).

## Study 1

### Method

#### Participants

Participants were recruited in three independent waves, with each wave completing one round of ratings. Wave one consisted of 25 participants, wave two consisted of 26 participants, and wave three consisted of 33 participants. Based on our a priori exclusion criteria, all participants were between the ages of 18–45 years old, were born in the United States, and self-identified as Black. In total, 84 Amazon Mechanical Turk workers who met these criteria participated in exchange for between $2.80 and $5.80, depending on the number of trials in the rating wave in which they participated. We restricted the sample to only Black participants in order to ensure the stimuli were distinctively perceived as Black and to eliminate any potential effects resulting from the race of the experimenter who created the stimuli (Asian) or other cross-race face processing differences.

#### Creation of stimuli

In order to create a BRME task that was equated with the original RME task, one of the authors collected images of male and female Black faces portraying different emotional states from publicly available online media. This is similar to the original RME task, which also used images of actors’ and actresses’ eyes as stimuli [1]. Faces were selected from scenes in which the character was portraying one of the 36 target mental states from the original task [1]. These images were converted to grayscale and cropped in Adobe Photoshop. All images had a resolution of 96 DPI. The eye region of each face (eyes, eyebrows, and bridge of the nose) was cropped using a rectangular area of 466 × 185 pixels, such that the stimuli were the same size as those used in the original version of the task [1].

The RME task and the BRME task both use the same 36 sets of words [1]. Each set of words included four answer choices, one of which correctly described the emotion shown by the eyes and three of which were distractors. As in the development of previous versions of the RME, the correct answer choice was determined by participant consensus (i.e., greater than 60% agreement) [1,2]. The location of the target word and distractor choices was identical to that in the Baron-Cohen et al. (2001) RME task [1]. The task was self-paced, although participants were encouraged to respond quickly. Prior to the task, participants were shown a screen with the following instructions (formatting included):

“You will be viewing images of eyes. For each set of eyes, choose which word best describes what the person in the picture is thinking or feeling. You may feel that more than one word is applicable but please choose just one word, the word which you consider to be most suitable.

Please make sure that you have read all 4 words before making your choice. The glossary of some of the words will be provided together with each set of words.

You should also try to give your response as fast as possible and do not overthink.

**Your ratings are very important to our research, so please choose the word that you think best describes what the person is thinking or feeling**.”

All target words and distractors are listed in S1 Table.

Based on the literature, the following selection criteria for our stimuli were used: at least 60% of participants chose the target word, and fewer than 25% of participants chose any single distractor word for each image [1,2]. Following the identification of the emotion participants were prompted to select the race of the target face (options: White, Black or African American, American Indian or Alaska Native, Asian, Native Hawaiian or Other Pacific Islander, or Other; Hispanic was included as an option in rounds two and three only). To ensure the faces were perceived to be Black, we only included face stimuli that were identified as Black by at least 84% of our participants and were identified as White a maximum of once. After the first round of ratings, we opted to keep six face stimuli that were rated as White by 1/17 participants (items: anticipating, desire, despondent, distrustful, fantasizing, and thoughtful). When these items were rated in the second round, 0/25 participants rated them as White. No other stimuli were ever rated as White in any of the three rounds of ratings.

One of the authors compiled a large pool of possible stimulus images; this pool was then narrowed down by removing stimuli that did not meet the selection criteria between each wave of ratings. The initial wave included 260 trials (one of which was the attention check trial). The second wave included 170 trials (eight of which were attention check trials). The final wave included 80 trials (eight of which were attention check trials). Between each iterative wave of ratings, stimuli that failed to meet the selection criteria were removed and replaced with new stimuli that were tested with the next wave of raters. This process continued until 36 final Black stimuli that met the predetermined selection criteria and provided us with the 36 mental states used in the original Baron-Cohen et al. (2001) stimulus set were obtained. When multiple possible stimuli fit the criteria for a given mental state, the stimulus that had more in common with the original Baron-Cohen et al. (2001) stimulus for that mental state was chosen (e.g. three possible stimuli fit the criteria for “insisting”; the only one that had direct eye gaze was selected because the Baron-Cohen “insisting” stimulus had direct eye gaze) [1]. If similarity to the Baron-Cohen et al. (2001) task was the same across multiple stimuli that all fit the criteria for a given mental state, the stimulus with higher resolution was selected.

Due to differences in the number of stimuli we could find portraying each emotion, the number of images tested for each item (i.e. for each of the 36 mental states) during each round of ratings varied. In the first two rounds, the minimum number of stimuli tested per item in any given round was two and the maximum number of stimuli tested per item was nine. By the third round of ratings, only eight items remained that did not have stimuli that had met all inclusion criteria during the previous two rounds. Of the remaining items requiring stimulus images, the minimum number of stimuli tested per item was one and the maximum number of stimuli tested per item was fourteen. For each mental state, each face was always paired with the same target and distractor words. Target and distractor words were taken directly from the original RME task in order to ensure the Black RME stimulus set was equated and compatible with the original White RME stimulus set [1].

Study 1 data are available at https://osf.io/2bd5a/.

## Results

### Data exclusions

To ensure a matched Black RME task, we collected multiple waves of stimulus piloting. Stimuli that did not equate with the original RME task [1] were eliminated and replaced in the next wave. In *wave one*, seven participants were excluded for failing the only attention check. For this attention check, participants were instructed to click a target word (“jealous”) while viewing a face that showed the emotion “panicked”; all seven participants who failed this check instead selected the correct answer choice (“panicked”) for the picture and not the required response “jealous”. To avoid this problem in later waves, we increased the number of attention checks to eight and required 75% accuracy (at least six out of eight correct), and all trials instructed participants to click the correct, given answer word for the target face (i.e. “For this face, please select panicked for your response” for a face that showed “panicked”). Additionally, one participant was also excluded in wave one because they were not born in the United States, resulting in a final sample size of 17 for wave one (${\bar{x}}_{\text{age}}=28.76$, *s* = 4.13, 9 female).

In *wave two*, with the modified attention check criteria, no participants were excluded for failing the attention checks. One participant was excluded because they identified as biracial, resulting in a final sample size of 25 for wave two (${\bar{x}}_{\text{age}}=31.12$, *s* = 7.43, 8 female).

In *wave three*, two participants were excluded for accuracy below 75% on the attention check trials. Seven additional participants who did not identify as Black were also removed, resulting in a final sample size of 24 for wave three (${\bar{x}}_{\text{age}}=29.38$, *s* = 5.70, 5 female). Thus, across all three waves, our final sample size was 66 (${\bar{x}}_{\text{age}}=29.88$, *s* = 6.09, 22 female).

After three waves of piloting, we obtained 36 Black face stimuli that met the selection criteria. Half of the stimuli were female, representing the same gender composition as the stimuli developed by Baron-Cohen et al. (2001) [1]. The itemwise ratings for the final stimuli are provided in Table 1. Because the stimuli were rated different numbers of times depending on how many waves they were included in, the *n* for each item in the Black RME is also given. The *n* for the White RME pilot testing was always 225 [1]. In the original task, forty stimuli were selected based on ratings by eight pilot raters (five out of eight chose the correct answer and no more than two out of eight chose any given foil) [1]. These forty stimuli were subsequently rated by 225 pilot testers (no demographic data available); stimuli that resulted in accurate ratings 50% of the time or less or in a specific incorrect answer choice being selected more than 25% of the time were removed. Four items were removed on the basis of these criteria, resulting in the final stimulus set containing 36 items. For a statistical comparison of the BRME and the original RME task, see S2 Table and S1 Text.

**Table 1:** Comparison of original (White) RME and Black RME items.

Mental state	White RME % correct	White RME % foil	Black RME % correct	Black RME % foil	Black RME *n*	Black RME % rated Black
accusing	78.7	12.0	64.0	16.0	25	92.0
anticipating	82.1	7.6	70.4	16.8	42	89.2^a^
cautious 1	84.9	8.9	83.2	9.9	42	92.1
cautious 2	79.6	11.1	73.4	12.8	42	94.1
concerned	79.9	10.3	71.4	17.8	42	90.1
confident	79.5	13.8	76.2	13.9	42	87.2
contemplative	72.9	14.7	76.0	16.0	25	92.0
decisive	74.7	12.9	66.7	16.7	24	95.8
defiant	83.6	8.9	80.0	12.0	25	100.0
desire	68.4	20.4	70.4	19.8	42	94.1^a^
despondent	73.8	12.0	73.3	14.8	42	91.2^a^
distrustful	85.8	6.7	65.4	21.8	42	91.1^a^
doubtful	72.9	16.0	75.0	12.5	24	95.8
fantasizing 1	86.7	6.2	84.2	7.9	42	92.1^a^
fantasizing 2	76.0	13.3	79.2	16.7	24	91.7
flirtatious	79.6	9.3	62.4	22.8	42	94.1
friendly	63.4	18.8	89.2	4.9	42	86.1
hostile	68.3	17.0	68.0	20.0	25	100.0
insisting	64.4	17.3	70.4	13.9	42	90.1
interested 1	88.0	6.7	84.0	8.0	25	92.0
interested 2	77.3	12.4	76.0	8.0	25	100.0
nervous	84.9	10.2	68.0	24.0	25	92.0
pensive	80.9	14.7	83.2	11.9	42	92.1
playful	75.6	12.4	77.3	14.8	42	86.1
preoccupied 1	64.9	21.8	66.5	17.8	42	87.2
preoccupied 2	72.9	19.6	71.4	17.8	42	91.2
reflective	64.4	21.8	79.2	8.3	24	95.8
regretful	65.8	22.2	88.0	8.0	25	96.0
serious	71.9	16.5	74.2	17.9	42	94.1
skeptical	90.2	4.4	70.4	9.9	42	95.1
suspicious	52.0	20.0	68.0	16.0	25	84.0
tentative	60.4	23.6	68.4	18.8	42	94.1
thoughtful	65.8	23.1	62.4	16.8	42	92.1^a^
uneasy	79.1	16.4	84.0	8.0	25	88.0
upset	73.3	10.7	61.5	17.8	42	89.2
worried	81.3	15.6	87.5	4.2	24	87.5

Item-by-item breakdown of the percent of participants who selected the target words and most popular distractor words (i.e. foil) for the original White RME stimuli [1] and the new Black RME stimuli.

^a^These items were rated as White by 1 participant in the first round of ratings. These items were never rated as White in subsequent rounds (i.e. 1/42 participants rated the item to be White); thus we opted to include these items in the final stimulus set.

### Discussion

We developed a 36-item RME task with Black mentalizing targets that was equated with the original RME task [1]. Henceforth we refer to this task as the Black Reading the Mind in the Eyes (BRME) test. The stimuli were clearly recognized as Black by our Black participants. In addition, the stimulus set had similar characteristics to the Baron-Cohen et al. (2001) stimulus set. Both sets of stimuli have the same gender composition (50% female), and both sets of stimuli depict the same target mental states to be inferred. Most importantly, participants chose the correct target word and the most popular distractor with equal frequency between both stimulus sets. Subsequent to piloting the BRME task, we examined whether the BRME task and the original RME task were equally difficult for both Black and White participants.

## Study 2

### Introduction

Because the 36-item BRME stimulus set developed in Study 1 was tested with only Black participants, we further piloted the stimuli to confirm that the BRME and the original RME tasks were equally difficult for non-Black participants. We used a 2 (participant race: Black, White) × 2 (target race: Black, White) between-subjects design to assess accuracy differences between the two stimulus sets. Specifically, we aimed to ensure there was no significant interaction between participant race and target race on performance accuracy, which could suggest that the difficulty between the two tasks was not equivalent for participants of different racial groups (e.g., White participants may be more accurate on the original RME task compared with the BRME task). Equating the difficulty of the tasks would allow researchers to assess individual differences that contribute to disparities in intergroup mentalizing without the concern that differences in accuracy may simply reflect task difficulty differences.

### Method

#### Participants

One-hundred forty Black (*n* = 70) and White (*n* = 70) participants (${\bar{x}}_{\text{age}}=29.92$, *s* = 9.21, 51 female (one participant did not report gender)) were recruited from the University of Chicago Center for Decision Research and the University of Chicago Downtown Research Lab. All participants were born in the United States, were between the ages of 18 and 50 years old, and self-identified as either Black or White per our a priori inclusion criteria. Participants were compensated at a rate of $1 per 5 minutes for their time.

#### Stimuli

The 36-item stimulus set developed by Baron-Cohen and colleagues was used for the White mentalizing task [1]. The BRME task developed in Study 1 was used for the Black mentalizing task.

#### Protocol

Participants completed a brief pre-test demographic survey to confirm they met the a priori inclusion criteria for this study. Only participants who met the demographic inclusion criteria continued with the protocol. These participants were randomly assigned to complete either the original RME task with exclusively White stimuli or the BRME task with exclusively Black stimuli (between subjects). Each task consisted of 36 trials presented in randomized order. Demographic questions were asked again after the RME task to confirm their initial pre-test demographics. The experimental task was run on Inquisit Web Version 4.0 (https://www.millisecond.com/), and both pre- and post-test demographics were collected on Qualtrics (https://www.qualtrics.com/).

#### Data analysis

We used mixed-effects logistic regression to analyze these data with the lme4 package (version 1.1–21) [50] in the R programming language (version 3.4.3) [51] on a Mac computer running OS X version 10.11.6. The dependent variable was trial accuracy (0 = incorrect and 1 = correct). The between-subjects factors were target race, which was contrast coded such that -0.5 denoted Black targets and 0.5 denoted White targets, participant race, which was contrast coded such that -0.5 denoted Black participants and 0.5 denoted White participants, and participant gender, which was contrast coded such that -0.5 denoted male participants and 0.5 denoted female participants. We allowed for between-subjects variance in intercepts to account for variations in response accuracy.

Study 2 data are available at https://osf.io/9v3yn/.

## Results

Importantly, none of the factors involving target race (i.e. the two separate stimulus sets) significantly predicted participant accuracy (see Table 2). The main effect of target race was not significant, nor were the interactions involving target race (target race × participant race, target race × participant gender, and target race × participant race × participant gender) (see Table 2). Including participant gender did not impact the results of the study (i.e., the target race × participant race did not emerge when removing participant gender).

**Table 2:** RME response accuracy model.

Fixed effects:					
	*b* Estimate	*SE*	CI_95%_	*z*-value	*p*-value
Intercept	1.009	0.064	[0.858, 1.117]	15.724	< 0.001 *
Target race	-0.066	0.125	[-0.185, 0.320]	-0.527	0.598
Participant race	0.369	0.128	[0.130, 0.646]	2.885	0.004 *
Participant gender	0.079	0.126	[-0.150, 0.355]	0.625	0.532
Target race × participant race	0.217	0.251	[-0.718, 0.291]	0.866	0.386
Target race × participant gender	0.237	0.257	[-0.738, 0.291]	0.922	0.356
Participant race × participant gender	-0.449	0.252	[-0.988, 0.021]	-1.785	0.074
Target race × participant race × participant gender	0.358	0.513	[-1.510, 0.549]	0.698	0.485

Logistic regression results on response accuracy in the RME task indicating influences of target race and participant race and gender. Asterisks indicate significant results.

We found a significant main effect of participant race such that White participants (${\bar{x}}_{\text{accuracy}}=0.750$, *s* = 0.433) were significantly more accurate than Black participants (${\bar{x}}_{\text{accuracy}}=0.672$, *s* = 0.469), *regardless* of the race of the mentalizing target (*b* = 0.427, *SE* = 0.126, CI_95%_ = [0.180, 0.673], *z* = 3.394, *p* < 0.001). However, this main effect did not involve differences between the stimulus sets, as supported by the nonsignificant two-way interaction of participant race and target race (see Table 3). Therefore, we did not find evidence suggesting differences in difficulty between the RME and the BRME task.

**Table 3:** Descriptive statistics for Black and White participants on the Black and White RME tasks.

	**Black RME proportion correct**	**White RME proportion correct**
**Black participants**	= 0.689, *s* = 0.463, *n* = 39	= 0.651, *s* = 0.477, *n* = 31
**White participants**	= 0.755, *s* = 0.430, *n* = 35	= 0.745, *s* = 0.436, *n* = 35

Cell means, standard deviations, and sample sizes for the non-significant target race × participant race interaction. Note that although White participants were significantly more accurate than Black participants at both RME tasks, there were no significant effects involving target race, suggesting both stimulus sets are similarly difficult for both racial groups tested.

### Discussion

Accuracy on the tasks did not differ as a function of the race of the mentalizing targets. Neither the target race main effect nor any interactions involving target race significantly predicted accuracy, suggesting that inferring mental states from the Black RME task is similarly difficult as it is from the White RME task developed by Baron-Cohen and colleagues [1].

## General discussion

We developed an RME task using Black target faces that was equated with the original all-White RME task [1]. In Study 1, Black raters confirmed the mentalizing targets appeared to be Black, and they chose the target words and most popular distractors with the same frequency as did the raters who were used to develop other versions of the RME test [1,2]. In Study 2, target race did not influence Black and White participants’ mentalizing accuracy. Results from Studies 1 and 2 indicate that the BRME task can be used either alone or in conjunction with the original White RME task developed by Baron-Cohen and colleagues (2001) in future mentalizing research. The present results are fully consistent with the two sets of mentalizing targets being compatible in mentalizing difficulty. Thus, we have created a stimulus set that will enable researchers to test mentalizing accuracy from a more diverse pool of perceptual targets.

Study 2’s aim to ensure no significant difference between participant race and target race on performance accuracy may seem surprising given previous work on the denial of secondary emotional states to outgroup members (e.g., [52], for review see [53]). Because the current work focused on accuracy at inferring secondary emotions (i.e., participants must choose one of four secondary emotions to attribute to the target), and not the tendency to attribute secondary emotions in the first place, we did not base the current predictions in that literature. This is a worthwhile future direction to determine how group membership influences accuracy of inferring secondary emotions. The BRME stimulus set can facilitate research in this domain.

In Study 2, we observed that overall, Black participants were less accurate than White participants; however, this difference did not vary as a function of target race. While we are confident this difference does not reflect differences in the difficulty of the task given that target race influenced mentalizing accuracy similarly for Black and White participants, the participant race effect was nonetheless surprising. It is possible that Black and White participants differed on other factors (e.g., education, socioeconomic status) that could influence RME performance, however, this data was not collected as part of this pilot study. Using a sample that is 50% Black is not typical for research that has used the RME task to date, and it is possible that results from these previous studies do not generalize to more diverse participant populations; this is an important question for future work. This research is unable to differentiate these possibilities, which warrant future research specifically designed and powered to detect such individual difference effects.

Additional important future directions can be gleaned from the extensive literature on the biological factors that influence performance on the RME test. Although an extensive review of this literature is beyond the scope of the present work, we briefly highlight major findings from this research. First, studies have identified a link between increased oxytocin and RME accuracy among both neurotypical adult men [19] and male teenagers and young adults (ages 12–19 years old) with autism spectrum disorders [54]. Second, RME performance appears to be dependent on normal amygdala and orbitofrontal cortex function. Individuals with bilateral amygdala lesions and individuals with bilateral orbitofrontal lesions were both significantly less accurate at inferring complex mental states from the eyes than were healthy controls [21,55]. Thus, there appears to be some biological basis for RME performance that future research should explore with this more diverse mentalizing task.

Previous research has also found a small but significant gender difference in RME accuracy between men and women (*d* = 0.21) such that women were more accurate than men [56]. Results suggested a genetic basis for this difference. Among women, RME accuracy was associated with a specific single-nucleotide polymorphism, whereas among men this association was absent [56]. In line with this gender difference in RME accuracy, testosterone has been negatively associated with RME performance. For example, fetal testosterone levels are negatively correlated with 6–8 year old children’s RME scores [57], and women were significantly less accurate at the RME test following testosterone administration than they were following placebo administration [58]. We did not observe a significant participant gender effect. However, given that previous RME studies have not typically included a large proportion of Black participants, it is possible that something about participant race may moderate the RME accuracy gender differences. This is an intriguing possibility that future research should investigate.

### Accessing the BRME stimuli

We provide unrestricted access to the BRME stimulus set (36 Black stimuli) and a key specifying all answer choices and their corresponding on-screen locations (see S2 Text). To access the BRME stimuli, interested users may submit a form available on our lab website (http://ifsnlab.org/). Access will be limited to research purposes only. Additionally, we will track the number of downloads and users’ reported purposes for downloading the stimuli. Following form submission, users will be automatically redirected to a download link for a zipped file containing all of the BRME stimuli and the aforementioned answer key. A variable guide for Study 1 is also included in this zipped file.

In future work, we aim to develop a database of RME tasks using targets from additional racial groups. Such a database would have a number of potential uses for social cognition researchers, particularly those interested in theory of mind and the influence of race on social cognitive processes. This BRME task represents an important first step toward the development of such a database. A more diverse stimulus set is valuable in and of itself in the sense that it increases the generalizability of any results drawn from the task; however, the BRME task will specifically enable researchers to investigate how Black perceivers mentalize from same-race perceptual targets. Given psychology’s historical dependence on samples of White undergraduate students, expanding research in a meaningful way to include subjects who have been traditionally ignored by our discipline is an important endeavor [59]. The BRME task will allow researchers to more effectively study diverse participant populations within the context of theory of mind and mentalizing from visual perceptual cues.

## Supporting information

S1 Table RME target words and distractors. Complete list of all 36 sets of target words and distractor choices.
(DOCX)

S2 Table Overall descriptive statistics for target words and distractors. Overall percent of participants who selected the target words and most popular distractor words for the original White RME stimuli [1] and the new Black RME stimuli. Note that the White RME stimuli were rated by Baron-Cohen and colleagues’ (2001) sample (participant race not reported) and the Black RME stimuli were rated by the Black participants in Study 1 of the present work.
(DOCX)

S1 Text Statistical comparison of average target and most popular distractor accuracy between BRME and original RME tasks. Independent samples *t*-tests showed that overall, the percentages of judges who selected the target word and the most popular distractor word for the Black stimuli did not significantly differ from the percentage of judges who selected the target word and the most popular distractor word for the White stimuli in Baron-Cohen and colleagues (2001) sample [1], (*t*(35) = 0.552, *p* = 0.584, CI_95%_ = [-2.609, 4.559] for target words, and *t*(35) = -0.131, *p* = 0.896, CI_95%_ = [-2.608, 2.291] for the most popular distractor words; see S2 Table).
(DOCX)

S2 Text BRME download instructions. All 36 BRME stimuli and their associated target words are available to download for academic use from our lab website (http://ifsnlab.org/). A “Read Me” text file is also included with the BRME stimulus set download. This supplemental text is the verbatim text from the Read Me file that is included with the download file containing all BRME stimuli.
(DOCX)
