# Supplementary material for: Black “Reading the Mind in the Eyes” task: The development of a task assessing mentalizing from black faces
Source: PLoS One. 2019 Sep 19;14(9):e0221867. doi: 10.1371/journal.pone.0221867 (PMC6752818; doi:10.1371/journal.pone.0221867)
Supplement: S1 Table — Complete list of all 36 sets of target words and distractor choices. (DOCX) [file pone.0221867.s001.docx]

**S1 Table.  RME Target Words and Distractors.**

| Target Word | Distractor #1 | Distractor #2 | Distractor #3 |
| --- | --- | --- | --- |
| accusing | irritated | disappointed | depressed |
| anticipating | decisive | threatening | shy |
| cautious (1) | insisting | bored | aghast |
| cautious (2) | joking | arrogant | reassuring |
| concerned | embarrassed | guilty | fantasizing |
| confident | ashamed | joking | dispirited |
| contemplative | flustered | encouraging | amused |
| decisive | amused | aghast | bored |
| defiant | contented | apologetic | curious |
| desire | joking | flustered | convinced |
| despondent | relieved | shy | excited |
| distrustful | aghast | baffled | terrified |
| doubtful | affectionate | playful | aghast |
| fantasizing (1) | aghast | impatient | alarmed |
| fantasizing (2) | embarrassed | confused | panicked |
| flirtatious | grateful | hostile | disappointed |
| friendly | dominant | guilty | horrified |
| hostile | alarmed | shy | anxious |
| insisting | joking | amused | relaxed |
| interested (1) | panicked | incredulous | despondent |
| interested (2) | joking | affectionate | contented |
| nervous | puzzled | insisting | contemplative |
| pensive | irritated | excited | hostile |
| playful | comforting | irritated | bored |
| preoccupied (1) | annoyed | hostile | horrified |
| preoccupied (2) | grateful | insisting | imploring |
| reflective | impatient | aghast | irritated |
| regretful | terrified | amused | flirtatious |
| serious | ashamed | bewildered | alarmed |
| skeptical | indifferent | embarrassed | dispirited |
| suspicious | ashamed | nervous | indecisive |
| tentative | arrogant | grateful | sarcastic |
| thoughtful | irritated | encouraging | sympathetic |
| uneasy | apologetic | friendly | dispirited |
| upset | terrified | arrogant | annoyed |
| worried | irritated | sarcastic | friendly |

Complete list of all 36 sets of target words and distractor choices.

1.     Baron-Cohen S, Wheelwright S, Hill J, Raste Y, Plumb I. The “Reading the Mind in the Eyes” test revised version: A study with normal adults, and adults with Asperger syndrome or high‐functioning autism. J Child Psychol Psychiatry. 2001;42: 241–251. doi:10.1111/1469-7610.00715
