# Supplementary material for: Black “Reading the Mind in the Eyes” task: The development of a task assessing mentalizing from black faces
Source: PLoS One. 2019 Sep 19;14(9):e0221867. doi: 10.1371/journal.pone.0221867 (PMC6752818; doi:10.1371/journal.pone.0221867)
Supplement: S2 Table — Overall percent of participants who selected the target words and most popular distractor words for the original White RME stimuli [1] and the new Black RME stimuli. Note that the White RME stimuli were rated by Baron-Cohen and colleagues’ (2001) sample (participant race not reported) and the Black RME stimuli were rated by the Black participants in Study 1 of the present work. (DOCX) [file pone.0221867.s002.docx]

**S2 Table. Overall Descriptive Statistics for Target Words and Distractors.**

|  | **Target word** | | | | **Most popular distractor word** | | | |
| --- | --- | --- | --- | --- | --- | --- | --- | --- |
|  | Mean | *SE* | Min. | Max. | Mean | *SE* | Min. | Max. |
| Black RME stimuli | 74.133 | 1.294 | 61.5 | 89.2 | 14.306 | 0.837 | 4.2 | 24.0 |
| White RME stimuli | 75.108 | 1.453 | 52.0 | 90.2 | 14.147 | 0.878 | 4.4 | 23.6 |

Overall percent of participants who selected the target words and most popular distractor words for the original White RME stimuli [1] and the new Black RME stimuli. Note that the White RME stimuli were rated by Baron-Cohen and colleagues’ (2001) sample (participant race not reported) and the Black RME stimuli were rated by the Black participants in Study 1 of the present work.

1.     Baron-Cohen S, Wheelwright S, Hill J, Raste Y, Plumb I. The “Reading the Mind in the Eyes” test revised version: A study with normal adults, and adults with Asperger syndrome or high‐functioning autism. J Child Psychol Psychiatry. 2001;42: 241–251. doi:10.1111/1469-7610.00715
