# Supplementary material for: Black “Reading the Mind in the Eyes” task: The development of a task assessing mentalizing from black faces
Source: PLoS One. 2019 Sep 19;14(9):e0221867. doi: 10.1371/journal.pone.0221867 (PMC6752818; doi:10.1371/journal.pone.0221867)
Supplement: S1 Text — Independent samples t-tests showed that overall, the percentages of judges who selected the target word and the most popular distractor word for the Black stimuli did not significantly differ from the percentage of judges who selected the target word and the most popular distractor word for the White stimuli in Baron-Cohen and colleagues (2001) sample [1], (t(35) = 0.552, p = 0.584, CI95% = [-2.609, 4.559] for target words, and t(35) = -0.131, p = 0.896, CI95% = [-2.608, 2.291] for the most popular distractor words; see S2 Table). (DOCX) [file pone.0221867.s003.docx]

**S1 Text. Statistical Comparison of Average Target and Most Popular Distractor Accuracy between BRME and Original RME Tasks.**

Independent samples *t*-tests showed that overall, the percentages of judges who selected the target word and the most popular distractor word for the Black stimuli did not significantly differ from the percentage of judges who selected the target word and the most popular distractor word for the White stimuli in Baron-Cohen and colleagues (2001) sample [1], (*t*(35) = 0.552, *p* = 0.584, CI_95%_ = [-2.609, 4.559] for target words, and *t*(35) = -0.131, *p* = 0.896, CI_95%_ = [-2.608, 2.291] for the most popular distractor words; see S2 Table).

1.     Baron-Cohen S, Wheelwright S, Hill J, Raste Y, Plumb I. The “Reading the Mind in the Eyes” test revised version: A study with normal adults, and adults with Asperger syndrome or high‐functioning autism. J Child Psychol Psychiatry. 2001;42: 241–251. doi:10.1111/1469-7610.00715
