# Supplementary material for: Black “Reading the Mind in the Eyes” task: The development of a task assessing mentalizing from black faces
Source: PLoS One. 2019 Sep 19;14(9):e0221867. doi: 10.1371/journal.pone.0221867 (PMC6752818; doi:10.1371/journal.pone.0221867)
Supplement: S2 Text — All 36 BRME stimuli and their associated target words are available to download for academic use from our lab website (http://ifsnlab.org/). A “Read Me” text file is also included with the BRME stimulus set download. This supplemental text is the verbatim text from the Read Me file that is included with the download file containing all BRME stimuli. (DOCX) [file pone.0221867.s004.docx]

**S2 Text.  BRME Download Instructions.**

All 36 Black Reading the Mind in the Eyes (BRME) stimuli and their associated target words are available to download for academic use from our lab website (http://ifsnlab.org/). A data file including all equating data from Study 1 and a “Read Me” text file are also included with the BRME stimulus set download.

The following text is included in the Read Me file (readme.txt) that is included with the download file containing all BRME stimuli.

READ ME

**********

RME TASK GUIDE

**********

Each stimulus is presented only once per participant. The stimulus is always presented with a given set of answer choices (see Answer Choices sheet in BRME_key.xlsx). Presentation order of the mental states is randomized.

Task Instructions:

You will be viewing images of eyes. For each set of eyes, choose which word

best describes what the person in the picture is thinking or feeling. You

may feel that more than one word is applicable but please choose just one

word, the word which you consider to be most suitable.

 Please make sure that you have read all 4 words before making your choice.

The glossary of some of the words will be provided together with each set of

words.

 You should also try to give your response as fast as possible and do not

overthink.

 Your ratings are very important to our research, so please choose the word

that you think best describes what the person is thinking or feeling.

List of All Mental States:

accusing

anticipating

cautious 1

cautious 2

concerned

confident

contemplative

decisive

defiant

desire

despondent

distrustful

doubtful
fantasizing 1
fantasizing 2
flirtatious
friendly
hostile
insisting
interested 1
interested 2
nervous
pensive
playful
preoccupied 1
preoccupied 2
reflective
regretful
serious
skeptical
suspicious
tentative
thoughtful
uneasy
upset
worried

**********

GUIDE TO BRME_key.xlsx SHEETS

**********

(Sheet 1) Answer Choices:

Lists each mental state and its corresponding answer choices. Column headers denote the on-screen location of each answer choice. Correct responses are bolded.

(Sheet 2) Screen Positions:

Provides the on-screen positions used in the BRME task, set in percentages to accommodate different screen sizes. Horizontal position is relative to the left side of the screen, with 0% representing the screen’s left edge and 100% representing the screen’s right edge. Vertical position is relative to the top of the screen, with 0% representing the screen’s top edge and 100% representing the screen’s bottom edge.

(Sheet 3) Study 1 Equating Data:

Equating data for the BRME and Baron-Cohen et al. (2001) RME stimulus sets. See Variable Guide below.

**********

VARIABLE GUIDE FOR STUDY 1 EQUATING DATA

**********

Mental state:

Label of the correct target mental state.

BRME n:

Number of participants who rated each individual item in the final BRME stimulus set (varies due to differences in the number of rounds each item was included in).

Note: The n for the White RME pilot testing was always 225 (Baron-Cohen et al., 2001).

BRME % target:

The total number of participants who selected the target mental state divided by the total number of participants who provided answers for that stimulus item (i.e. BRME n) multiplied by 100.

BRME % foil:

The total number of participants who selected the most popular distractor answer choice divided by the total number of participants who provided answers for that stimulus item (i.e. BRME n) multiplied by 100.

BRME % rated Black:

The total number of participants who identified the stimulus face as “Black or African American” divided by the total number of participants who rated that stimulus item (i.e. BRME n) multiplied by 100.

BRME % rated White:

The total number of participants who identified the stimulus face as “White” divided by the total number of participants who rated that stimulus item (i.e. BRME n) multiplied by 100.

Baron-Cohen et al. (2001) RME % target:

The total number of participants who selected the target mental state divided by the total number of participants who provided answers for that stimulus item (n = 255) multiplied by 100.

Baron-Cohen et al. (2001) RME % foil:

The total number of participants who selected the most popular distractor answer choice divided by the total number of participants who provided answers for that stimulus item (n = 255) multiplied by 100.

1.     Baron-Cohen S, Wheelwright S, Hill J, Raste Y, Plumb I. The “Reading the Mind in the Eyes” test revised version: A study with normal adults, and adults with Asperger syndrome or high‐functioning autism. J Child Psychol Psychiatry. 2001;42: 241–251. doi:10.1111/1469-7610.00715
